# Medical Lexicon of Modern Terminology

**Published:** 1849-04

**Authors:** 


*Medical Lexicon of Modern Terminology.* Being a complete vocabulary of definitions, including all the technical terms employed by writers and teachers of medical science, at the present day, &c. Second edition, greatly enlarged, by D. Meredith Reese, M. D., LL. D., resident physician of Bellevue Hospital, N. Y., &c. New York: Samuel S. and Wm. Wood, 1849, pp. 240, 18mo.

We have received from the publishers, the second edition of the above named work. Soon after the first was issued from the press, we took occasion to speak of it in terms of high commendation. The second has been considerably enlarged and improved, thus, very greatly enhancing its value, and increasing its claims to the attention of the student and practitioner of medicine. We believe it contains all the terms used in modern medical literature, with accurate and concise definitions. In the preparation of this dictionary, Dr. Reese has rendered a valuable service to the medical student and practitioner. It would also be found equally valuable to the student and practitioner of dental surgery. It is brought out in a very neat and convenient form, and will be found to be a valuable pocket companion.

